# Confronting climate change in the age of denial

**DOI:** 10.1371/journal.pbio.3000033

**Published:** 2018-10-09

**Authors:** Liza Gross

**Affiliations:** Public Library of Science, San Francisco, California, United States of America

## Abstract

This Editorial introduces a Collection of articles in which the authors explore the challenges and pitfalls of communicating the science of climate change in an atmosphere where evidence doesn't matter.

This Editorial is part of the *Confronting Climate Change in the Age of Denial Collection*.

What is it about stories that captures the human imagination? Writing coaches will tell you that readers care more about what happens next than they do about beautiful prose. Neuroscientist Michael Gazzaniga says that stories help us make sense of the world. In the 1970s, Gazzaniga identified a brain region in the left hemisphere, which he dubbed “the interpreter,” that tries to fit everything into a story, even filling in missing gaps, in a deep-seated need to create order from chaos [1]. Trouble is, our reliance on story to interpret the world also makes us vulnerable to conspiracy theories and false narratives that happen to dovetail with our values and worldview. Scientists and science educators have long wrestled with the challenges of communicating evidence that contradicts people’s personal, religious, or political beliefs, particularly regarding evolution, vaccine safety, and climate change.

A perfect case study of people’s tendency to create their own narratives in the face of incomplete information is the recent viral response to a photo of a starving polar bear. The photographers intended to show what the future of climate change might look like: polar bears depend on sea ice to hunt seals, walruses, and other prey. As more sea ice melts, bears will lose their hunting platforms and likely starve to death, placing the species’ survival at risk. But people interpreted the image through their own personal lens. Some recognized the effort to illuminate the costs of climate change while others seized on the photo to deny climate change by pointing to healthy bears as evidence that the species is doing fine. The photographers recently said they were shocked by the response and realized they’d “lost control of the narrative” [2].

Against this backdrop, we asked an Arctic mammal expert and two social scientists to weigh in on the challenges and pitfalls of communicating scientific evidence around climate change. Their contributions appear in the collection publishing this week, “Confronting climate change in the age of denial” [3].

Sue Moore and Randall Reeves draw on decades of research on marine mammals to set the record straight on the likely impacts of climate change on wildlife populations in the Arctic. “Marine mammals are ecosystem sentinels, capable of reflecting ocean variability through changes in their ecology and body condition,” Moore, a biological oceanographer, and Reeves, a marine mammal biologist, write in “Tracking arctic marine mammal resilience in an era of rapid ecosystem alteration” [4].

They explore the life history traits of endemic and migratory Arctic species to gauge their capacity to adapt to ecosystem changes caused by rapid warming and identify potential winners (bowhead and gray whales) and losers (polar bears and walruses). The authors propose a framework to facilitate rapid assessments of population status and guide management and conservation efforts. The framework enhances traditional approaches, they argue, by including ecological indicators—including geographic range and behavior—and physiological indicators, in addition to traditional demographics, to provide a more comprehensive view of the health of populations. This Arctic marine mammal tracking network, they say, could feed into global ocean surveys, which ultimately “offer a path toward sustainability through improved prediction, more precaution, and wiser policy in this era of global environmental change”.

In “Climate communication for biologists: When a picture can tell a thousand words” [5], psychologists Stephan Lewandowsky and Lorraine Whitmarsh examine strategies for using the anecdotes and images that satisfy our need for narrative without sacrificing scientific accuracy.

“This basic human tendency to rely on anecdotes, stories, and recent experiences presents a particular dilemma in relation to climate change,” write Lewandowsky and Whitmarsh. “Perhaps more than most other scientific facts, the evidence for climate change is based on statistical analyses of innumerable observations that are dispersed across time and space. It takes up to 17 years of data to reliably detect a warming trend, and the large variability of the weather that is superimposed on that inexorable trend always provides an opportunity to point to some location on Earth that is experiencing record-breaking cold or snowfall, thereby providing an anecdote that, in people’s minds, may overpower the overwhelming scientific evidence that the globe is warming”.

Likewise, environmental activists may deploy images that can evoke powerful emotional responses. These images may be broadly accurate but can also mislead by tying climate change to specific events, such as the death of an individual polar bear or the severity of a raging firestorm. And denialists may well jump on these fudges to spin patently false narratives about climate change. Yet it’s possible to overcome these issues, Lewandowsky and Whitmarsh argue, by figuring out how people can become emotionally engaged with climate change and identifying legitimate triggers for those responses.

Science communication experts Michael Dahlstrom and Dietram Scheufele explore another dimension of the peril and promise of using stories to communicate science in “(Escaping) the paradox of scientific storytelling” [6]. Our tendency to remember events and interpret new information—not on its own merits but by trying to force it into our already established narratives—runs counter to the way scientists are trained, Dahlstrom and Scheufele argue. Scientists often see stories as unscientific and manipulative. And though storytelling is “agnostic to truth” and science communicators must compete with storytellers who don’t care about evidence, they say, there is still a place for story in science.

Rather than telling stories to simply impart knowledge—which may prove unsuccessful, they say, since increased scientific literacy does not lead to greater acceptance of science—it may be better to tell stories about how scientific knowledge is produced. “In the end, using storytelling to primarily build scientific support through knowledge, attitude, or behavior goals without also engaging scientific reasoning might not help science in the long run”.

Most scientists consider climate change one of the greatest challenges of our time—if not the greatest. That’s why *PLOS Medicine* commissioned a special issue last year to explore the diverse health impacts associated with climate change [7] and PLOS created an online “Channel” in which experts highlight the latest research, news, and commentaries on the effects of climate change [8].

There is overwhelming scientific consensus that climate change is real and that we’re driving it (see Fig 1) [9]. Still, even though our failure to curb greenhouse gases could transform life as we know it, just 48% of United States adults believe the scientific consensus [10].

**Fig 1:**
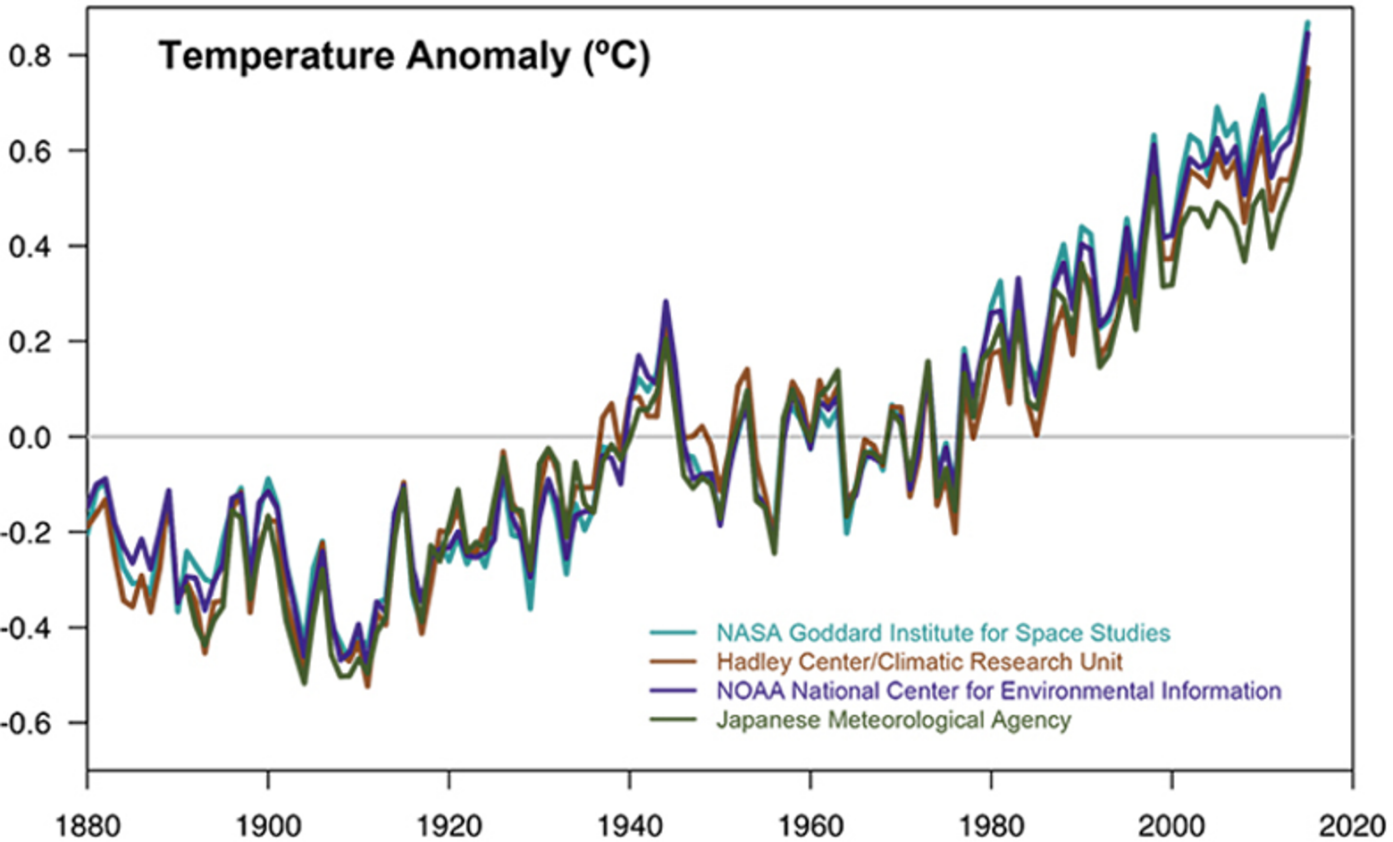
This graph aggregates temperature data from four institutions to show rapid warming in the past few decades, with the last decade the warmest on record. Data sources: NASA's Goddard Institute for Space Studies, National Oceanic and Atmospheric Administration National Climatic Data Center, Met Office Hadley Centre/Climatic Research Unit, and the Japanese Meteorological Agency. *Graph courtesy NASA*.

As the authors in our collection make clear, part of this gap can be explained by the human tendency to shoehorn new information into pre-existing narratives. But powerful vested interests that are intent on denying inconvenient evidence also play a role. Scores of studies have revealed the sophisticated strategies deployed by the tobacco industry to deny overwhelming evidence that smoking and secondhand smoke cause cancer and heart disease [11]. Scholars and journalists have since documented similar duplicitous disinformation campaigns waged by the chemical and fossil fuel industries. Following the tobacco industry playbook, they rely on third-party allies who feign independence while promoting false narratives to help their clients delay regulations and protect their bottom line.

Our penchant for storytelling, anthropologists believe, transcends culture, time, and place. Stories once helped us survive by providing a way for communities to share information about where to hunt, how to avoid danger, and where to find shelter. The fairy tales we hear as children offer life lessons about persistence, greed, sacrifice, heroes, and villains. As you read the pieces in this collection, consider the range of actors competing for people’s attention through this ancient mode of communication. Some are using stories that relay scientific information to help people make sense of the world. Others are using stories that manipulate science to serve their own interests. Following the tobacco industry’s lead, Exxon orchestrated a climate change denial campaign that stalled meaningful efforts to reduce greenhouse gases for decades [12].

We hope that everyone who values unbiased scientific evidence thinks about ways to harness storytelling to help people grasp this complex but very real threat to our planet. We need to reclaim the storyline before it’s too late.
